# Complete Functional Recovery of a Feline with Extensive Facial Injuries Following a Traffic Accident

**DOI:** 10.3390/ani15081161

**Published:** 2025-04-17

**Authors:** Seung-Hyun Kim, Manbok Jeong, Yeong-Bin Baek, Jang-Han Yoon, Jun-Gyu Park, Sang-Ik Park

**Affiliations:** 1 Department of Veterinary Surgery, College of Veterinary Medicine, Chonnam National University, Gwangju 61186, Republic of Korea; trazet08@gmail.com; 2 KSH Surgical Animal Medical Center, Gwangju 61617, Republic of Korea; tomcoma1@gmail.com; 3 Laboratory of Veterinary Ophthalmology, College of Veterinary Medicine, Chonnam National University, Gwangju 61186, Republic of Korea; mbjeong@jnu.ac.kr; 4 Department of Veterinary Pathology, College of Veterinary Medicine, Chonnam National University, Gwangju 61186, Republic of Korea; ybbaek@jnu.ac.kr; 5 Department of Veterinary Zoonotic Diseases, College of Veterinary Medicine, Chonnam National University, Gwangju 61186, Republic of Korea; 6 BK21 FOUR Program, Department of Veterinary Pathology, College of Veterinary Medicine, Chonnam National University, Gwangju 61186, Republic of Korea

**Keywords:** multiple complex fractures, temporomandibular joint, mandibular fracture, hard palate fracture, orbital fracture, traffic accident, multiple surgical interventions

## Abstract

This report examines the successful recovery of a feline that presented with multiple complex fractures and dislocations involving the facial and cranial structures resulting from a traffic accident. Diagnostic CT imaging identified significant injuries, including luxation of the left temporomandibular joint (TMJ), a mandibular symphyseal fracture, a hard palate fracture, and a left orbital fracture accompanied by severe exudate within the nasal cavity, compressing the left orbit and nasal passages. Importantly, no additional injuries were detected in the thoracic or abdominal regions, facilitating a more targeted treatment plan. The management of this case required extensive surgical intervention, including open reduction of the TMJ, stabilization of the mandibular symphysis, repair of the bony palate, and partial maxillectomy. After 20 days of ICU hospitalization, the feline fully recovered. This outcome is particularly noteworthy as the combination of severe injuries observed in this case is unprecedented in the veterinary literature. Consequently, it offers critical insights into both surgical techniques and postoperative management strategies applicable to similarly complex trauma cases. The feline’s full recovery, characterized by the restoration of normal daily functions, highlights the clinical significance of pursuing multiple, complex surgical procedures in cases of severe trauma. It serves as a valuable reference for advancing the understanding and management of severe facial trauma in veterinary practice.

## Simple Summary

This case report details the remarkable recovery of a cat that suffered severe craniofacial injuries from a traffic accident. The feline presented with multiple fractures, including a dislocated jaw, a fractured palate, and an orbital fracture, leading to respiratory distress. Diagnostic imaging confirmed the extent of the injuries, prompting immediate surgical intervention, which involved temporomandibular joint reduction, mandibular stabilization, bony palate repair, and partial maxillectomy. After 20 days in intensive care, the cat achieved full functional recovery, regaining the ability to eat and move normally. This case is significant as it highlights successful treatment strategies for complex maxillofacial trauma in veterinary medicine, offering valuable insights into advanced surgical techniques and postoperative care.

## 1. Introduction

Trauma is a common cause of injury in cats, with the head being one of the most frequently affected regions [1,2]. Among trauma-related fractures, up to 27% involve the skull, with facial fractures accounting for approximately 69% of these cases, making them a significant consequence of traffic accidents [2,3,4,5,6,7,8].

The diagnosis of maxillofacial injuries in traumatized cats can be challenging due to soft tissue swelling and the limited accessibility of deep facial regions through physical examination alone [9]. Clinical manifestations may include epistaxis, abrasions, ocular trauma, jaw fractures, symphyseal separation, dental fractures, and temporomandibular joint (TMJ) luxations [9,10]. The accurate localization of facial fractures is critical for developing effective treatment strategies. Advanced diagnostic imaging, particularly computed tomography (CT), is considered superior to plain radiography for the detailed assessment of craniomaxillofacial anatomical structures and fracture patterns [9,11].

Surgical management of facial fractures involves a range of techniques, including plate fixation, interfragmentary wiring, mandibular cerclage, external skeletal fixation, and mandibulectomy [12,13,14,15]. TMJ injuries may be treated using methods such as maxillomandibular fixation, conservative management, or reduction in luxations through either closed or open approaches [16].

In this case, the feline presented with multiple complex facial and cranial injuries, including fractures and luxation. Surgical intervention involved repairs to the TMJ, mandibular symphysis, and bony palate, as well as a maxillectomy. Postoperatively, the cat received 20 days of intensive care unit (ICU) hospitalization, resulting in a full functional recovery. This case represents a unique and unprecedented combination of extensive facial injuries in the veterinary literature. It provides valuable insights into the surgical and postoperative management of severe craniofacial trauma, emphasizing the importance of comprehensive, multidisciplinary approaches. The successful restoration of the cat’s daily functional abilities underscores the clinical significance of pursuing complex surgical interventions, even in cases of severe trauma, as they can lead to highly favorable outcomes.

## 2. Case Description

A 5-year-old neutered male cat was referred to a regional surgical center following a car accident, with radiographic findings confirming severe facial trauma. Clinical presentation included severe epistaxis, eyeball protrusion, significant facial swelling, moderate dyspnea, and loss of consciousness. Immediate hospitalization in the ICU with high-flow oxygen support was required. Emergency diagnostics, including ultrasonography, radiography, and laboratory tests (CBC, biochemistry, and electrolytes), were conducted promptly.

Laboratory analysis indicated a severe inflammatory response, with feline serum amyloid A measured at 165.6 µg/mL (reference range: 5–10 µg/mL). Imaging ruled out thoracic or abdominal trauma but confirmed extensive facial injuries, including fractures and luxation. Based on the owner’s strong desire to save the cat, immediate emergency surgery was pursued. CT scans were performed to confirm the diagnosis and refine the surgical plan. However, the patient’s respiratory function progressively declined due to hemorrhagic exudates in the nasal cavity, necessitating mechanical ventilation and a carefully tailored anesthesia plan.

A 22-gauge intravenous (IV) catheter was placed in the right cephalic vein, and pre-anesthetic medication included remifentanil (1 µg/kg, IV). After 2 min of preoxygenation, anesthesia was induced with alfaxalone (2 mg/kg to effect, IV). Intubation was achieved using a 4 mm internal diameter endotracheal tube, inflated to prevent air leaks at 20 cm H_2_O. The patient was maintained on a rebreathing system with oxygen at 1 L/min and isoflurane (1%) in combination with remifentanil constant rate infusion at 0.2 µg/kg/min. Pressure-regulated volume control ventilation (PRVC) was employed, with settings optimized to achieve a tidal volume of 10 mL/kg of predicted body weight and partial pressure of end-tidal CO_2_ between 35 and 45 mmHg.

**Figure 1:**
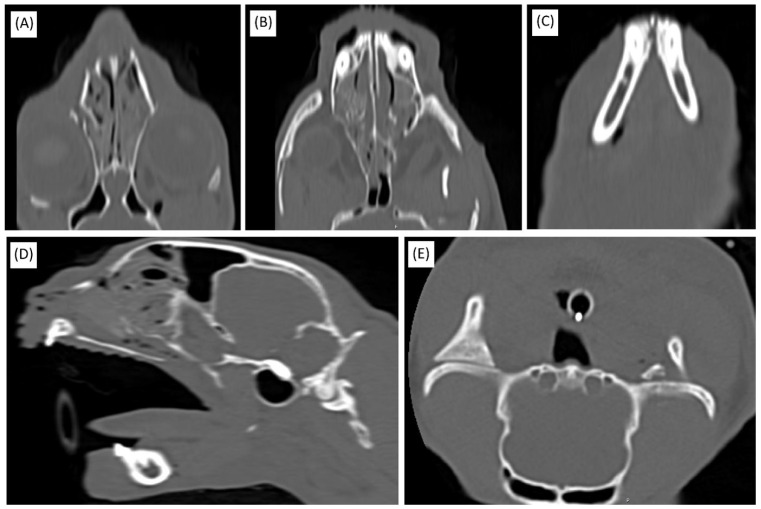
Diagnostic CT imaging of the skull. (**A**) Coronal view displaying the left maxilla/orbit fracture. (**B**) Coronal view highlighting the bony palate fracture. (**C**) Coronal view showing the mandibular symphysis fracture. (**D**) Sagittal view illustrating hemorrhagic exudate within the nasal cavity. (**E**) Axial view depicting the left temporomandibular joint (TMJ) luxation.

Considering the presence of multiple surgical sites and the patient’s critical condition—characterized by loss of consciousness and respiratory failure—optimized surgical strategies were adopted to minimize the procedural time and prevent further deterioration. Although time-consuming, computed tomography (CT) imaging was deemed indispensable for the accurate evaluation of craniofacial trauma and the formulation of a precise surgical plan. CT imaging confirmed extensive injuries involving multiple fractures and soft tissue compromise, necessitating prompt surgical intervention (Figure 1). Fortunately, no damage to individual teeth was identified upon visual inspection, palpation, or CT imaging.

**Figure 2:**
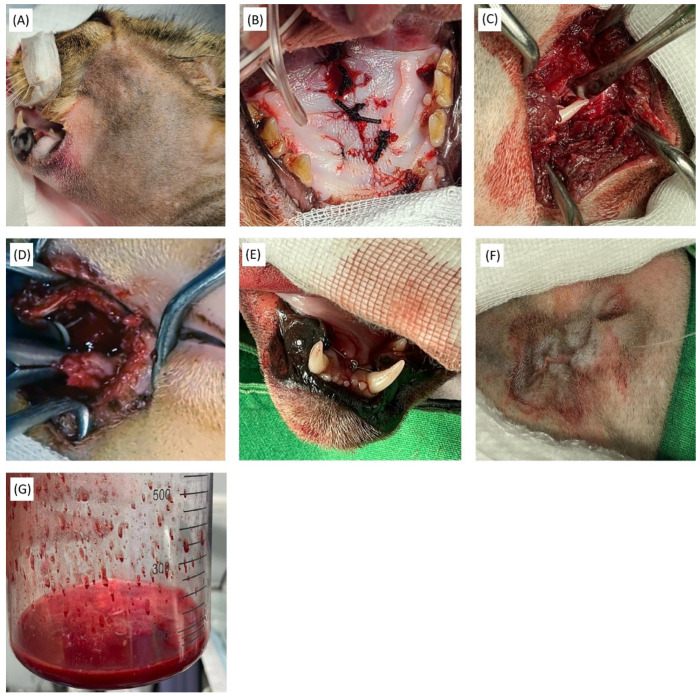
Multiple surgical interventions. (**A**) Preparation for surgery with preoperative disinfection. (**B**) Repair of bony palate fracture using non-absorbable braided silk suture. (**C**) Open reduction in left TMJ luxation. (**D**) Maxillectomy performed with removal of fractured bone using rongeur. (**E**) Stabilization of mandibular symphysis fracture with circumferential stainless-steel wiring. (**F**) Intradermal suture pattern applied for skin closure. (**G**) Lavage and suctioning of intranasal hemorrhagic exudates using medical suction machine.

Given the severity of the injuries and the owner’s strong desire for maximal recovery, the following surgical procedures were performed (Figure 2):

1. Mandibular symphysis repair: The mandibular symphyseal fractures were classified as non-complex. Therefore, a cerclage wire technique was selected. A circumferential stainless-steel wire was placed behind the canine teeth to achieve stable fixation.
2. Palatal fracture repair: Owing to the limited size of the palatal defect, a suture-based method was considered sufficient. To maintain tension during healing, non-absorbable braided silk sutures were used to reattach the defect.
3. TMJ luxation reduction: Closed reduction was initially attempted but was unsuccessful due to repeated failure. Consequently, open reduction was performed and proved successful, obviating the need for the planned contingency procedure, condylectomy.
4. Maxillectomy: A displaced bone fragment from the left maxillary/orbital fracture was causing nasal hemorrhage and damage to the left globe. While enucleation followed by orbital access would have been optimal in the presence of significant ocular injury, the owner strongly preferred globe preservation. Thus, maxillectomy was performed to access and remove the fragment without enucleation. Enucleation was prepared as a contingency in case of intraoperative complications or failure of the selected approach.
5. Among all procedures, the maxillectomy was the most technically challenging and high-risk. Intraoperative hemorrhage posed a serious threat due to the confined surgical field and the patient’s compromised status. To mitigate this risk, the infraorbital and major palatine arteries were preemptively ligated and transected using a Thunderbeat vessel-sealing device (Olympus, Tokyo, Japan). These vessels were selected based on their anatomical relevance and the minimal adverse effects expected from their ligation. The bone fragment was successfully removed using a rongeur.
6. Hemorrhage control: Suture ligation with synthetic absorbable monofilament sutures and a vessel-sealing device ensured hemostasis.

All nasal cavity exudates were carefully suctioned, and the left orbit’s compression was relieved. Intraoperative monitoring showed stable vital signs, and ventilation was transitioned to synchronized intermittent mandatory ventilation (PRVC-SIMV) postoperatively. The patient regained spontaneous respiration within 10 min of discontinuing isoflurane and was transferred to the ICU for continued care.

The patient remained hospitalized in the ICU for 20 days. Postoperative management included intravenous antibiotics (piperacillin/tazobactam, metronidazole, and moxifloxacin), opioid analgesia (hydromorphone), and a balanced crystalloid solution. Nutrition was provided via an esophagostomy tube until normal appetite and TMJ function were restored. Nasal cavity hemorrhage resolved within two days, and by day 10, the patient demonstrated spontaneous oral feeding and normal TMJ function. Although vision in the left eye was permanently lost, its protrusion and associated damage were resolved by day 14. By day 20, the cat achieved complete functional recovery and was discharged.

This case highlights the successful management of severe craniofacial trauma through comprehensive surgical intervention and meticulous postoperative care. Despite the complexity of the injuries and the need for multiple surgical procedures, the patient’s recovery underscores the potential for favorable outcomes when advanced diagnostics, tailored anesthesia, and multidisciplinary treatment strategies are employed. This case also contributes valuable insights to the veterinary literature, offering guidance for managing similarly severe trauma in small animal practice.

## 3. Discussion

Trauma is a frequent cause of injury in cats, and the head is one of the areas most often affected [1,2]. Fractures of various skull bones are frequently observed following head trauma, with studies reporting that up to 27% of trauma-related fractures involve the skull [2,3,7]. Among these, approximately 69% are associated with facial fractures [8]. Mandibular, maxillary, and craniofacial fractures occur in approximately 70% of cases, with TMJ injuries being the most common. In contrast, caudal skull fractures are relatively rare, affecting only 9% of cats [2,9].

Mandibular fractures, including mandibular symphyseal fractures, are the most frequently reported, accounting for 79–86% of cases [4,9]. Additionally, fractures in the mid-face region—such as those involving the nasopharynx (68.9%), orbit (68.9%), nose (51.1%), upper jaw (51.1%), intermaxillary suture (44.4%), and zygomatic arch (33.3%)—are also prevalent [9]. These statistics underscore the complexity of craniofacial trauma in feline patients and the challenges they present in clinical management.

As described earlier, this case involved a rare and complex constellation of craniofacial injuries that posed significant challenges to both structural reconstruction and functional recovery. Clinical complications included compression of the left orbit and nasal cavity, globe protrusion, and progressive accumulation of hemorrhagic intranasal exudates. These factors contributed to marked dyspnea, complicating anesthetic management and necessitating urgent surgical intervention.

The patient presented with a Modified Glasgow Coma Scale (MGCS) score of 7 and an Animal Trauma Triage (ATT) score of 11, distributed as follows: perfusion (1), cardiac (1), respiratory (3), eye/muscle/integument (2), skeletal (2), and neurologic function (2). According to a retrospective study evaluating 114 cases of feline craniofacial trauma [17], the survival-to-discharge rate was 47.4% in cases that underwent surgery, provided the patient did not meet grave prognostic criteria warranting euthanasia. Notably, only 32.3% of cats presenting with altered mentation at initial evaluation survived to discharge. Several poor prognostic indicators were identified in that study, including intact male status, vehicular- or animal-related trauma, lower MGCS scores, higher ATT scores, and altered mentation [17].

In the present case, the patient exhibited multiple negative prognostic indicators noted in the referenced study: a low MGCS score, a high ATT score, altered mentation, and a history of vehicular trauma [17,18]. Additionally, three of the four criteria associated with grave prognosis—including ocular changes, maxillary fractures, and altered mentation—were also present. Despite fulfilling the majority of these poor prognostic criteria, the patient achieved full recovery following timely and aggressive surgical intervention, highlighting the exceptional nature of the treatment and management provided.

The antibiotic regimen consisted of a triple-therapy approach: a beta-lactam/beta-lactamase inhibitor combination for broad-spectrum coverage, a quinolone for extended Gram-negative activity, and metronidazole for anaerobic coverage. In our region, amoxicillin–clavulanate and marbofloxacin are commonly used in small animal practice. However, due to the inability to confirm the patient’s prior medication history and the high risk of infection, we selected higher-tier antibiotics—piperacillin–tazobactam and moxifloxacin—while considering the potential for antimicrobial resistance.

A bacterial culture and antimicrobial susceptibility test were performed using a nasal blood sample, although the results required over five days to be returned. During this interval, empirical antibiotic therapy was continued. To monitor infection and inflammatory status, complete blood counts (CBCs) and feline serum amyloid A (fSAA) levels were assessed every other day. If two consecutive results had shown elevated inflammatory markers, escalation to meropenem would have been considered. Fortunately, the administered antibiotics were effective, and sequential test results demonstrated progressive improvement.

Postoperative complications associated with skull fractures in cats include dyspnea, TMJ ankylosis, impaired mastication, dental malocclusion, soft tissue edema, implant failure, damage to neurovascular structures, oronasal fistulas, osteomyelitis, and regurgitation [2,12,14,16]. Specifically, complications related to jaw fractures may result in malocclusion, delayed union or non-union of fractures, dental trauma, or difficulty eating [2].

In this case, meticulous surgical techniques and comprehensive ICU management helped to mitigate these risks. Postoperative care included the placement of a central venous catheter and an esophagostomy tube, which facilitated fluid therapy, nutritional support, and effective medication delivery. Despite the severity of the injuries, no significant postoperative complications were observed, and the patient achieved full functional recovery.

Timely surgical intervention also played a critical role in the successful outcome. The patient arrived at our facility approximately one hour after the traumatic event, and diagnostic imaging followed by emergency surgery was completed within three hours of presentation. Based on the trauma classification system described in the literature [19], the patient fell into the category of “patients requiring immediate surgery to prevent imminent death”. This categorization was supported by progressive respiratory deterioration due to intranasal hemorrhage, which necessitated life-saving surgical intervention. Consequently, preoperative resuscitation and stabilization were intentionally minimized to avoid any delay.

However, despite our best efforts, several limitations were encountered in this case. Although modern reconstructive technologies—such as CT-based 3D reconstruction and 3D-printed titanium implants—have significantly advanced the field of complex maxillofacial surgery, we were unable to implement these techniques for the following reasons. First, limitations in image data quality and technical resources, combined with the urgent nature of the case, precluded the generation of high-resolution 3D reconstructions. Second, in our region, CT-based 3D printing typically requires a fabrication and delivery period of at least one week. While this may be feasible for elective orthopedic or reconstructive procedures, it is not appropriate for urgent surgical cases such as this, where time is a critical factor.

Had these technologies been readily available, the precision and completeness of the surgical reconstruction could have been significantly enhanced—a limitation we acknowledge with regret. We hope that, as such resources become more accessible, other clinicians will consider incorporating these advanced modalities into their surgical planning and execution.

To our knowledge, this is the first documented case in the veterinary literature describing such a unique combination of severe craniofacial injuries that resulted in a successful long-term outcome. The patient has remained healthy for over two years postoperatively, without any notable complications, thereby underscoring the effectiveness of the interventions employed.

This case holds substantial clinical value, demonstrating that the successful management of extensive feline craniofacial trauma is achievable through a combination of precise surgical techniques and intensive postoperative care. While the surgical methods utilized may not be novel in themselves, their integrated application under emergency conditions and in a highly complex context is noteworthy. This report emphasizes the importance of a multidisciplinary approach and highlights the pivotal role of surgical expertise in achieving favorable outcomes. Ultimately, the findings contribute meaningfully to the growing body of veterinary surgical knowledge and may serve as a practical reference for clinicians managing similarly complex trauma cases in the future.

## 4. Conclusions

This case report underscores the importance of a multidisciplinary approach in managing severe craniofacial trauma in veterinary medicine. The feline patient presented with a rare combination of complex fractures, including TMJ luxation, mandibular symphyseal separation, hard palate disruption, and maxillary/orbital fractures—injuries that are typically associated with significant functional impairment and a guarded prognosis. Advanced imaging techniques, particularly CT, played an essential role in facilitating precise surgical planning, enabling targeted interventions to restore structural integrity and function.

The treatment strategy emphasized a combination of reconstructive procedures, including TMJ realignment, mandibular stabilization, and partial maxillectomy, complemented by intensive postoperative care. The successful outcome—characterized by the restoration of normal feeding behavior and functional recovery within 20 days—highlights the potential for favorable prognoses even in cases of extensive maxillofacial trauma. Despite the complexity of the injuries, the absence of major postoperative complications reinforces the efficacy of a well-coordinated surgical and critical care approach.

This case contributes to the growing body of veterinary literature on maxillofacial trauma, demonstrating that even in cases involving severe structural damage, functional rehabilitation is achievable with early intervention and evidence-based surgical techniques. Given that craniofacial fractures account for a significant proportion of trauma-related injuries in feline patients, further studies should focus on optimizing surgical protocols and evaluating long-term outcomes to refine clinical guidelines for similar cases.

## Data Availability

All data are contained within the article.
